# Rapid assessment of disability in the Philippines: understanding prevalence, well-being, and access to the community for people with disabilities to inform the W-DARE project

**DOI:** 10.1186/s12963-016-0096-y

**Published:** 2016-08-02

**Authors:** Manjula Marella, Alexandra Devine, Graeme Ferdinand Armecin, Jerome Zayas, Ma Jesusa Marco, Cathy Vaughan

**Affiliations:** 1 Nossal Institute for Global Health, The University of Melbourne, Level 4, Alan Gilbert Building, 161 Barry Street, Carlton, VIC 3010 Melbourne, Australia; 2 Social Development Research Centre, De La Salle University, Manila, Philippines; 3 Centre for Health Equity, Melbourne School of Population and Global Health, The University of Melbourne, Melbourne, Australia

**Keywords:** Disability, Prevalence, Philippines, Participation, Risk factors, Survey

## Abstract

**Background:**

International recognition that people with disabilities were excluded from the Millennium Development Goals has led to better inclusion of people with disabilities in the recently agreed Global Goals for Sustainable Development (SDGs) 2015–2030. Given the current global agenda for disability inclusion, it is crucial to increase the understanding of the situation of people with disabilities in the Philippines. The aim of this study was to estimate the prevalence of disability and compare the well-being and access to the community between people with and without disabilities.

**Methods:**

A population-based survey was undertaken in District 2 of Quezon City and in Ligao City. 60 clusters of 50 people aged 18 years and older were selected with probability proportion to size sampling from both locations. The Rapid Assessment of Disability (RAD) survey was used to identify people with disabilities based on their responses to activity limitations. The levels of well-being and access to the community for people with disabilities were compared with controls matched by age, gender, and cluster. Information on barriers to accessing the community was also collected.

**Results:**

The prevalence of disability was 6.8 (95 % CI: 5.9, 7.9) and 13.6 % (95 % CI: 11.4, 16.2) in Quezon City and Ligao City respectively. Psychological distress was the most commonly reported condition in both locations, although it was often reported with a co-morbid condition related to sensory, physical, cognitive, and communication difficulties. The prevalence of disability was associated with age and no schooling, but not associated with poverty. People with disabilities had significantly lower well-being scores and reduced access to health services, work, rehabilitation, education, government social welfare, and disaster management than people without disability. Having a disability and negative family attitudes were reported as barriers for people with disabilities participating in work, community meetings, religious activities, and social activities.

**Conclusions:**

The prevalence of disability among adults in District 2 of Quezon City and in Ligao City is higher than the estimated national prevalence of disability derived from the 2010 Philippines census. Disability is also associated with lower well-being and reduced participation across a number of domains of community life.

## Background

People with disabilities face all forms of discrimination and exclusion from the social, cultural, political, and economic life of their communities [1]. In many contexts, people with disabilities are more likely to experience poverty, yet are often not appropriately considered or included in development programs [2–5]. There is growing recognition of the need for disability-disaggregated data to increase understanding of the prevalence of disability and, equally importantly from a development programming perspective, to support the design, implementation, and monitoring of effective, inclusive development programs [1]. Responding to the needs and priorities of people with disabilities, for example, requires context-specific information on the lived experience of disability and how this intersects with access to essential life domains, such as health, education, employment, and social inclusion.

The Women with Disability taking Action on Reproductive and sexual health project (referred to hereafter as W-DARE), is a three-year participatory action research project [6]. The aim of W-DARE is to improve access to quality sexual and reproductive health for women with disability in the Philippines, and has comprised three distinct research phases. In line with the participatory action research approach, the findings from each phase informed the design and activities of the subsequent phase. Phase one involved the collection of quantitative and qualitative data to understand the lived experience of people with disabilities in the Philippines. This included a cross-sectional household survey using the Rapid Assessment of Disability (RAD), in two districts of the Philippines. This paper outlines the quantitative findings from this survey that aimed to (1) determine the prevalence of disability and its socioeconomic correlates in people aged 18 years and above in District 2 of Quezon City and in Ligao City; and (2) compare well-being and participation of people with disabilities in their community to those without disability in the two locations.

The Philippines is an emerging market economy and one of the world’s most populated countries with a population of 92.3 million people [7]. The Philippines was ranked 117 out of 187 countries in the 2013 Human Development Index [8]. Similar to many lower- and middle-income countries, the population is young with a median age of 23.4 years, and highly urbanized with 45.3% of people now living in urban areas [7]. Non-communicable diseases such as cardiovascular diseases, cancer, chronic obstructive pulmonary disease, and diabetes are on the rise and are a major cause of mortality and morbidity in the Philippines [9]. There are numerous and wide-ranging estimates of the prevalence of disability in the Philippines, ranging from 1.6 as estimated in the 2010 Census of Population and Housing [10], to 28.2 % according to the World Report on Disability [1]. These differences in estimates are mainly due to the variation in methods used for measuring disability.

The Philippines ratified the United Nations Convention on the Rights of Persons with Disabilities (CRPD) [11] in 2008, and several laws and policies to promote the rights of people with disabilities have been enacted [12]. However, a study commissioned by Disability Rights Promotion International (DRPI) and the National Federation of Organizations of people with disabilities in the Philippines (Katipunan ng Maykapansanan sa Pilipinas, Inc., KAMPI) in 2008, found that a number of the rights of people with disabilities were regularly violated [12]. The study interviewed people with disabilities from Metro Manila, and the Luzon, Mindanao, and Visayas island groups. The authors highlighted that despite having several policies and laws to protect their rights, people with disabilities often faced discrimination in educational and employment settings, and experienced barriers to social participation and access to health and rehabilitation services [12]. The study recommended a set of immediate measures to eliminate barriers to participation and for the economic empowerment of people with disabilities. However, socioeconomic factors associated with disability and the level of access to services and participation in the community compared to people without disability were not studied.

International recognition that people with disabilities were excluded from the Millennium Development Goals has led to better inclusion of people with disabilities in the development of the recently agreed Global Goals for Sustainable Development (SDGs) 2015–2030, with disability explicitly mentioned in 5 of the 17 goals, particularly Target 17, which focuses on disability data disaggregation [13]. In line with Article 31 of the CRPD, which charges State Parties to collect data on disability to inform disability-inclusive policies [11], the SDGs will include targets for collecting data specific to disability. Given the current global agenda for disability inclusion, it is crucial to increase understanding of the situation of people with disabilities in the Philippines. This includes generating reliable estimates on the prevalence of disability and data about the participation of people with disabilities in the community. Information on the level of participation and the impact of social and environmental factors on a person’s functioning are important for both the planning and monitoring of disability-inclusive policies and programs in the Philippines.

## Methods

A cross-sectional population-based survey was conducted in District 2 in Quezon City (a densely populated, urban district) and in Ligao City, Albay (a predominantly rural district) in the Philippines between December 2013 and January 2014. The RAD questionnaire was used in this study. The RAD survey was developed to measure different domains of disability described in the International Classification of Functioning and Disability (ICF) framework [14, 15]. The survey was validated in Bangladesh and Fiji [15].

### Study design and sampling

A sample size of 3,010 was required to estimate disability prevalence with 95 % confidence level, 20 sampling error, a design effect of 1.5, a non-response rate of 10, and a conservative estimate of prevalence of disability at 5 %. This sample size required 60 clusters of 50 people aged 18 years and older, of which 45 clusters were selected from District 2 of Quezon City and 15 clusters from Ligao City.

The clusters were selected with probability‐proportionate to size, separately for District 2 of Quezon City and Ligao City, using the updated data from the 2010 Census of Population and Housing [7] as the sampling frame. Households within clusters were selected through compact segment sampling where each cluster was divided into clearly demarcated segments of equal population of about 50 people aged 18 years and above. Detailed maps with landmarks and roads were obtained from the National Statistical Office or drawn. One segment from each cluster was randomly selected by drawing lots. Within the selected segment, the survey team visited all households door-to-door, until 50 people aged 18 years and older were recruited. A household was defined as a group of people who lived together, pooled their money, and ate at least one meal together each day. When an eligible household member was absent, at least two return visits were made. In the case of a sample of 50 people aged 18 years and older not being reached in a segment, households from another randomly selected segment were recruited.

### RAD questionnaire

The RAD questionnaire [15] is interviewer-administered and has two parts: a household questionnaire administered to the head of the household and an individual questionnaire administered to each individual in the household. The household questionnaire assesses household demographics and socioeconomic status based on household characteristics such as source of water, having electricity, sanitation facility, roof, wall and floor materials, plus asset indicators including durable goods (e.g., television, radio, bicycle and motorcycle), and ownership of the house and land.

The individual questionnaire comprises four sections: 1) demographics, 2) self-assessment of functioning, 3) well-being, and 4) access to the community. The demographic section includes items related to age, gender, ethnicity, religion, marital status, education, occupation, health conditions, and information on any assistive devices used. The self-assessment of functioning section includes items related to functioning in eight domains: vision, hearing, communication, mobility, gross and fine motor, cognitive, appearance, and psychological distress. Each item asks the participants to report the frequency of difficulty in functioning because of a health problem in the last 6 months even when using assistive devices available to them (e.g., seeing even if wearing glasses). The response categories are ‘none,’ ‘some of the time,’ ‘most of the time,’ and ‘all of the time.’ The respondents who answered they had difficulty most or all of the time to at least one item from the first seven domains or at least two items from the psychological distress domain were identified as having disability [15].

The well-being section includes items such as good health, making friends, being safe in daily life, and taking care of one’s self, where the frequency of experiencing the situation was reported on a 4-point Likert scale ranging from ‘never’ to ‘all of the time.’ The last section on access to the community is comprised of domains related to health, education, work, social, legal, religious, rehabilitation, and other services. Each domain has three items. The first item asks for the level of access to services with responses recorded on a 4-point Likert scale (‘as much as needed’ to ‘not at all’), with an additional category ‘had not needed the services.’ The second item asks for barriers to accessing services using open-ended questions. If participant responses include more than one barrier, they are asked to rank the most limiting barrier in the third item [15].

### Training and field testing of the questionnaire

Field supervisors and data collectors were recruited based on their skills and previous experience. People with and without disability were recruited as data collectors and were trained for a week on disability inclusion, study design, recruitment of participants, administration of the RAD questionnaire, ethics in research, collecting survey data, data storage, and referral mechanisms for participants. Supervised field practice sessions were conducted as part of the training.

Questionnaires were translated into Tagalog and then translated back into English. They were also cognitively tested on a convenience sample of 14 participants with different disabilities to ensure a range of respondents understood the questions as intended and that their responses accurately reflected what was being asked.

### Questionnaire administration

The RAD household questionnaire was administered to the head of household and the individual questionnaire to all eligible participants in the household. Each eligible member of the household was administered the ‘demographics’ and ‘self-assessment of functioning’ sections of the RAD. Only those participants identified to have disability were invited to complete sections on well-being and access to the community. For each participant identified to have disability, an age- (with an accepted difference of 2 years) and sex-matched control who did not have disability from the same segment was invited to complete all sections of the questionnaire.

### Ethics, consent, and permissions

Ethics approvals were obtained from the University of Melbourne Human Research Ethics Committee, Australia (ethics ID 1339640) and the De La Salle University Ethics Committee, Philippines. In accordance with local practice in the Philippines, relevant barangay kapitans (equivalent to head of a village or ward) were informed about the study and their endorsement was sought to facilitate introduction into the community. The study was conducted in accordance with the tenets of the Declaration of Helsinki. All participants provided written or verbal informed consent. For participants who were not literate, the consent form was read to them and their verbal agreement was recorded by the interviewer in front of a witness. This protocol was approved by the ethics committees.

### Statistical analysis

Statistical analyses were performed using PASW Statistics 18 (PASW Statistics for Windows, SPSS Inc., Chicago, IL). Disability (present or absent), as measured using the self-assessment of functioning section, was the dependent variable. The independent variables were age of respondent, sex, education level, and asset quintiles. Age was grouped into five categories (18–24, 25–34, 35–44, 45–55, and 55 years and over), and education into four categories (no schooling, elementary, high school, and college/technical). Asset index was used as a proxy indicator for wealth status using principal components analysis on the data from the household questionnaire [16]. Individuals were ranked according to the asset index of the household in which they resided. The households were then divided into quintiles, with the first quintile representing the poorest in the sample, and the fifth quintile representing the wealthiest. Both univariate and multivariate (binary logistic regression) analyses were undertaken to assess the associations between sociodemographic characteristics and prevalence of disability. The reference groups were 18–24 years, male, fifth quintile, college/technical education. Confidence intervals (CI) for prevalence estimates and regression odds ratios were calculated with adjustment for clustering effects in the study design using the generalized estimating equation approach. Age and sex adjusted prevalence was derived using projected population estimates for 2014 as the reference standard.

Rasch analysis was used to derive person measures for the well-being section. Rasch analysis is a form of Item Response Theory, where ordinal ratings are transformed to estimates of interval measures. Andrich rating scale model was used with Winsteps (Ver 3.80) to perform Rasch analysis [17]. The resulting measures showed adequate psychometric properties and therefore the Rasch scores were used in subsequent analyses. For ease of interpretation the scores were rescaled to range from 0 to 100, where a high score represented better well-being.

Multivariate logistic and linear regression analyses were undertaken to identify differences in quality of life and access to the community between cases and controls. The matching of cases and controls was not complete, particularly among those aged 55 years and over, and therefore analyses were adjusted by the matching variables of age and sex.

## Results

In Quezon City, a total of 2,610 people were enumerated, of whom 2,139 (82.0 %) participated in the survey and 471 (18.0 %) were either unavailable or declined to participate in the study. In Ligao City, a total of 823 people were enumerated, of whom 765 (93.0 %) participated in the survey and 58 (7.0 %) were either unavailable or declined to participate in the study. The mean ± SD age of participants was 38.9 ± 14.3 years in Quezon City and 41.9 ± 16.5 years in Ligao City, and women were over-represented in both locations. Participants in Quezon City were comparatively younger, and had a higher level of education compared to participants in Ligao City. Table 1 summarizes the demographic and socioeconomic characteristics of the sample from the two locations.

**Table 1:** Socioeconomic correlates of prevalence of disability in Quezon City and Ligao City

	Quezon City				Ligao City			
	Total sample (*n* = 2287) n (%)	People with disabilities (*n* = 159) n (%)	Prevalence of disability (95 % CI)^a^	Age-sex adjusted OR (95 % CI)^b^	Total sample (*n* = 772) n (%)	People with disabilities (*n* = 110) n (%)	Prevalence of disability (95 % CI)^a^	Age-sex adjusted OR (95 % CI)^b^
Gender								
Male	733 (32.1)	41 (25.8)	5.3 (3.9, 7.2)	1	345 (44.7)	49 (44.1)	13.2 (10.1, 17.2)	1
Female	1554 (67.9)	118 (74.2)	7.5 (6.3, 9.0)	1.5 (1.0, 2.1)	427 (55.3)	62 (55.9)	13.9 (11.0, 17.5)	1.1 (0.7, 1.6)
Age (years)								
18–24	440 (19.2)	12 (7.5)	2.7 (1.6, 4.7)	1	141 (18.3)	11 (9.9)	7.7 (4.3, 13.4)	1
25–34	563 (24.6)	24 (15.1)	4.3 (2.9, 6.3)	1.6 (0.8, 3.2)	155 (20.1)	12(10.8)	7.7 (4.5, 13.1)	1.0 (0.4, 2.4)
35–44	491 (21.5)	31 (19.5)	6.5 (4.6, 9.1)	**2.5 (1.3, 4.9)**	158 (20.5)	9 (8.1)	5.7 (3.0, 10.5)	0.7 (0.3, 1.8)
45–54	437 (19.1)	48 (30.2)	11.0 (8.4, 14.3)	**4.4 (2.3, 8.4)**	139 (18.0)	29 (26.1)	21.0 (15.0, 28.6)	**3.2 (1.5, 6.7)**
≥ 55	356 (15.6)	44 (27.7)	12.7 (9.7, 16.7)	**5.2 (2.7, 10.1)**	179 (23.2)	50 (45.0)	27.7 (21.7, 34.7)	**4.6 (2.3, 9.2)**
Education								
No schooling	155 (6.8)	26 (16.1)	16.2 (11.2, 22.9)	**4.4 (2.5, 7.5)**	139 (18.0)	42 (38.2)	29.5 (22.5, 37.6)	**5.7 (2.5, 13.0)**
Elementary	618 (27.0)	53 (32.9)	8.3 (6.4, 10.8)	**2.0 (1.3, 3.2)**	363 (47.1)	51 (46.4)	13.5 (10.4, 17.4)	2.1 (0.9, 4.7)
High school	659 (28.8)	46 (28.6)	6.7 (5.0, 8.9)	**1.6 (1.1, 2.6)**	165 (21.4)	9 (8.2)	4.8 (2.5, 9.0)	0.7 (0.3, 1.9)
College/technical	854 (37.4)	36 (22.4)	4.3 (3.1, 5.9)	1	104 (13.5)	8 (7.3)	6.8 (3.4, 13.2)	1
Socioeconomic status								
Poorest quintile	412 (18.4)	36 (22.9)	8.9 (6.5, 12.3)	1.4 (0.8, 2.3)	140 (18.8)	23 (21.1)	16.1 (10.9, 23.3)	1.4 (0.7, 2.8)
Second quintile	462 (20.6)	23 (14.6)	4.7 (3.1, 7.0)	0.7 (0.4, 1.2)	137 (18.4)	21 (19.3)	13.7 (9.0, 20.1)	1.2 (0.6, 2.3)
Third quintile	443 (19.7)	30 (19.1)	6.2 (4.4, 8.8)	0.9 (0.5, 1.6)	143 (19.2)	20 (18.3)	13.8 (9.0, 20.1)	1.2 (0.6, 2.3)
Fourth quintile	474 (21.1)	37 (23.6)	7.4 (5.4, 10.0)	1.1 (0.7, 1.8)	160 (21.5)	25 (22.9)	14.5 (9.9, 20.7)	1.3 (0.7, 2.4)
Wealthiest quintile	454 (20.2)	31 (19.7)	6.7 (4.8, 9.5)	1	163 (21.9)	20 (18.3)	11.9 (7.7, 17.8)	1

Values in bold represent statistical significance at *p* < 0.05

^a^Adjusted for age and sex

^b^Adjusted for age, sex, education, and socio-economic status

### Disability prevalence

The prevalence of functional limitation was 7.2 (95 % CI: 6.2, 8.3) in Quezon City and 14.0 % (95 % CI: 11.5, 17.0) in Ligao City. Age and sex adjusted prevalence of functional limitation was 6.8 (95 % CI: 5.9, 7.9) and 13.6 % (95 % CI: 11.4, 16.2) in Quezon City and Ligao City respectively.

**Fig. 1:**
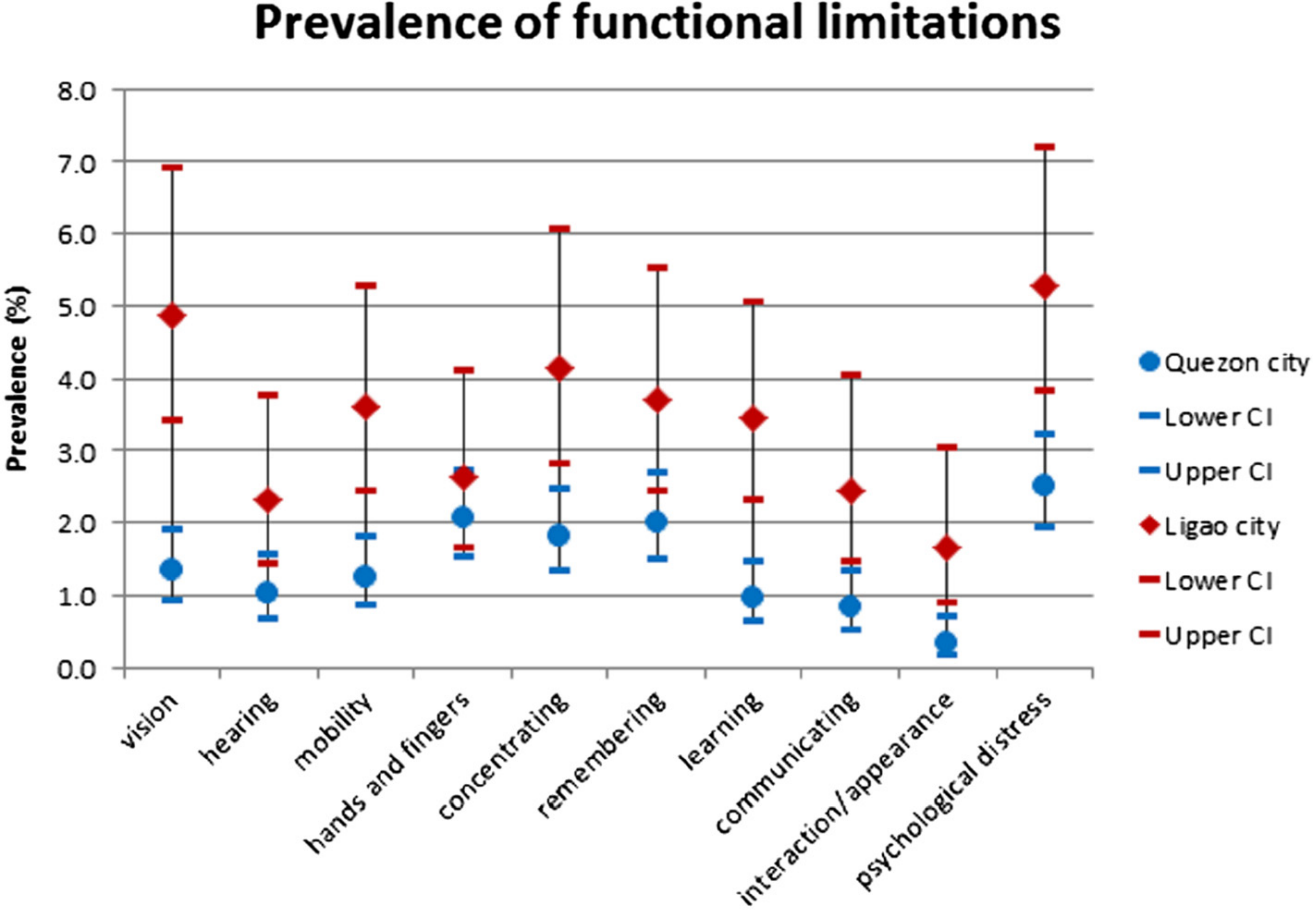
Prevalence of functional limitations in Quezon City and Ligao City

Psychological distress was the most commonly reported difficulty in the two samples: 2.5 (95 % CI: 1.9, 3.2) in Quezon City and 5.3 % (95 % CI: 3.8, 7.2) in Ligao City (Fig. 1). However, the majority of respondents (65 % in Quezon City and 78 % in Ligao City) with psychological distress also had co-morbid conditions related to sensory, physical, cognitive, and communication functional limitations. The prevalence of psychological distress alone (i.e., after excluding co-morbid functional limitations) was 0.9 (95 % CI: 0.5, 1.3) in Quezon City and 1.3 % (95 % CI: 0.6, 2.4) in Ligao City.

Excluding psychological distress, the most commonly reported difficulties with functioning were difficulty using hands and fingers (2.1, 95 % CI: 1.5, 2.7), remembering (2.0, 95 % CI: 1.5, 2.7), and concentrating (1.8 %, 95 % CI: 1.3, 2.5) in Quezon City. In Ligao City, seeing (4.9, 95 % CI: 3.4, 6.9), concentrating (4.2, 95 % CI: 2.8, 6.1), and remembering (3.7 %, 95 % CI: 2.5, 5.5) were the most commonly reported functional limitations.

Psychological distress was the most commonly reported difficulty for participants aged between 18 and 54 years in Quezon City and among the age groups 18 and 34 years, and 55 years and over in Ligao City. Physical difficulties related to using hands and fingers and mobility were the most commonly reported in 55 years and over age group in Quezon City. Seeing difficulty was the most commonly reported difficulty among participants aged between 35 and 54 years in Ligao City (Table 2).

**Table 2:** Most commonly reported difficulties in different age groups

Age groups, years	Quezon city	Ligao city
18–24	Psychological distress (4, 15.4 %)	Psychological distress (5, 20.8 %)
25–34	Psychological distress (10, 23.3 %)	Psychological distress (7, 26.9 %)
35–44	Psychological distress (12, 26.7 %)	Seeing (3, 27.3 %)
45–54	Psychological distress (20, 18.0 %)	Seeing (10, 17.2 %)
≥55	Hands and finger (13, 13.8 %) Mobility (13, 13.8 %)	Psychological distress (4, 15.4 %)

The prevalence of disability was higher among females than males in both locations, although the difference was not statistically significant (Table 1). In Quezon City, the prevalence of disability increased with age from 2.7 % (95 % CI: 1.6, 4.7) in 18–24 years age group to 12.7 % (95 % CI: 9.7, 16.7) in respondents 55 years and over. The prevalence of disability was not significantly different among people aged between 18 and 44 years in Ligao City. However, the prevalence increased considerably in the 45 years and over age groups, where one in five people aged 45–54 years and one in four people aged 55 years and over had a disability. In both locations the prevalence of disability was significantly higher among those with no schooling. Socioeconomic status was not related to disability in both locations.

### Comparison of well-being for cases and controls

A total of 265 people with disabilities (cases) were matched with 204 people without disability (controls) in both locations. Cases and controls were similar in age, sex, types of occupation, marital status, location, and socioeconomic status (Table 3). People with disabilities were significantly less likely to have schooling compared to people without disability.

**Table 3:** Socioeconomic characteristics of cases and controls

	Cases (*n* = 265) n (%)	Controls (*n* = 204) n (%)	Age-sex adjusted OR (95 % CI)
Gender			
Male	88 (33.2)	61 (29.9)	0.9 (0.6, 1.3)
Female	177 (66.8)	143 (70.1)	1
Age, years			
18–24	23 (8.7)	23 (11.3)	1
25–34	36 (13.6)	27 (13.2)	1.3 (0.6, 2.9)
35–44	39 (14.7)	37 (18.1)	1.1 (0.5, 2.2)
45–54	74 (27.9)	60 (29.4)	1.2 (0.6, 2.4)
> = 55	93 (35.1)	57 (27.9)	1.6 (0.8, 3.2)
Location			
Urban (Quezon City)	156 (58.9)	123 (60.3)	1
Rural (Ligao City)	109 (41.1)	81 (39.7)	1.0 (0.7, 1.5)
Level of education			
No schooling	66 (25.0)	36 (17.6)	2.0 (1.1, 3.6)
Elementary	102 (38.6)	81 (39.7)	1.5 (0.9, 2.4)
High school	53 (20.1)	36 (17.6)	1.7 (1.0, 3.2)
College/technical	43 (16.3)	51 (25.0)	1
Occupation			
Dependent	132 (50.8)	82 (41.4)	2.9 (0.7, 12.0)
Laborer/tradesman	71 (27.3)	57 (28.8)	2.2 (0.5, 9.5)
Farmer/skilled worker	54 (20.8)	53 (26.8)	1.7 (0.4, 7.1)
Professional/others	3 (1.2)	6 (3.0)	1
Current relationship			
Married/live in	181 (68.6)	146 (71.9)	1
Single/never married	83 (31.4)	57 (28.1)	1.2 (0.8, 1.8)
Socioeconomic status			
Poorest quintile	59 (22.8)	35 (18.1)	1.5 (0.8, 2.8)
Second quintile	41 (15.8)	41 (21.2)	0.9 (0.5, 1.6)
Third quintile	50 (19.3)	35 (18.1)	1.3 (0.7, 2.3)
Fourth quintile	60 (23.2)	41 (21.2)	1.2 (0.7, 2.2)
Wealthiest quintile	49 (18.9)	41 (21.2)	1

The well-being scores were significantly lower for people with disabilities (mean ± SD: 57.3 ± 14.6) compared to people without disability (mean ± SD: 66.1 ± 14.1), *p* < 0.001. The difference persisted after adjusting for age and sex.

### Comparison of access to community for cases and controls

Responses to the level of access to different domains of community were dichotomized with the positive response category ‘as much as needed’ coded as ‘met need’ and the three negative response categories as ‘unmet need’ (Table 4). People with disabilities generally had lower access to all domains in the community compared to people without disability. The difference was statistically significant for accessing health, work, rehabilitation, education, government social welfare, and disaster management between cases and controls. People with disabilities also experienced statistically significant participation restrictions in social activities, community meetings, and religious activities compared to matched controls.

**Table 4:** Access to the community between cases and controls

Domains	Cases n (%)	Controls n (%)	Age-sex adjusted OR (95 % CI)
Health			
Unmet need	80 (33.9)	35 (19.3)	1
Met need	156 (66.1)	146 (80.7)	0.5 (0.3, 0.7)
Work			
Unmet need	112 (45.0)	41 (21.5)	1
Met need	137 (55.0)	150 (78.5)	0.3 (0.2, 0.5)
Assistive devices			
Unmet need	51 (29.8)	17 (18.7)	1
Met need	120 (70.2)	74 (81.3)	0.6 (0.3, 1.1)
Rehabilitation			
Unmet need	72 (54.5)	19 (31.7)	1
Met need	60 (45.5)	41 (68.3)	0.4 (0.2, 0.7)
Social activities			
Unmet need	102 (55.1)	46 (30.9)	1
Met need	83 (44.9)	103 (69.1)	0.4 (0.2, 0.6)
Community meetings			
Unmet need	89 (38.7)	47 (25.1)	1
Met need	141 (61.3)	140 (74.9)	0.5 (0.3, 0.8)
Safe drinking water			
Unmet need	22 (8.3)	9 (4.5)	1
Met need	242 (91.7)	193 (95.5)	0.5 (0.2, 1.1)
Toilet facilities			
Unmet need	11 (4.2)	6 (3.0)	1
Met need	253 (95.8)	197 (97.0)	0.8 (0.3, 2.1)
Religious activities			
Unmet need	76 (30.4)	37 (19.1)	1
Met need	174 (69.6)	157 (80.9)	0.5 (0.3, 0.9)
Government social welfare services			
Unmet need	124 (51.0)	50 (30.9)	1
Met need	119 (49.0)	112 (69.1)	0.4 (0.3, 0.6)
DPO			
Unmet need	90 (90.9)	29 (70.7)	1
Met need	9 (9.1)	12 (29.3)	0.2 (0.1, 0.6)
Education			
Unmet need	120 (69.0)	55 (42.0)	1
Met need	54 (31.0)	76 (58.0)	0.3 (0.2, 0.5)
Disaster management			
Unmet need	56 (22.4)	22 (11.2)	1
Met need	194 (77.6)	174 (88.8)	0.4 (0.2, 0.7)
Legal services			
Unmet need	39 (34.8)	24 (28.6)	1
Met need	73 (65.2)	60 (71.4)	0.8 (0.4, 1.4)

The major barriers reported under each domain were similar among people with and without disabilities (Table 5). Lack of information and cost were the most commonly reported barrier for the majority of the domains. Barriers related to costs were both direct (fees), and indirect costs (e.g., transport). Disability was reported as a specific barrier by people with disabilities for participating in work, community meetings, and religious activities. People with disabilities also reported negative family attitudes, such as family not wanting them to participate in work and social activities, a barrier to community participation not reported by people without disability.

**Table 5:** Most limiting barriers for not accessing different domains in the community as much as needed among cases and controls

	Cases (n, %)	Controls (n, %)
Health	Costs (26, 34 %) Lack of information (16, 21 %)	Lack of information (10, 36 %) Costs (7, 25 %)
Work	Disability (38, 34 %) Family attitudes/taking care of family (27, 24 %)	Taking care of family (12, 29 %) Lack of opportunities (5, 12 %)
Assistive devices	Costs (29, 62 %) Lack of information (8, 17 %)	Costs (6, 60 %) Lack of information (3, 30 %)
Rehabilitation	Costs (22, 33 %) Lack of information (21, 32 %)	Lack of information (7, 41 %) Costs (6, 36 %)
Social activities	Family attitudes/taking care of family (21, 21 %) Costs (17, 17 %)	Costs (13, 28 %) Lack of information/events (22 %)
Community meetings	Busy with work or household (29, 32 %) Disability (15, 17 %)	Lack of information/no invitation (19, 45 %) Busy with work or household (5, 12 %)
Safe drinking water	Costs (9, 43 %) Lack of information (4, 19 %)	Lack of information (5, 56 %) Costs (2, 22 %)
Toilet facilities	Physical accessibility/No facilities (2, 30 %) Costs (2, 20 %)	No facilities (3, 50 %)
Religious activities	Lack of information/no invitation (18, 25 %) Disability (14, 19 %)	Lack of information/no invitation (12, 36 %) Costs (4, 12 %)
Government social welfare services	Lack of information (64, 54 %) No services (16, 14 %)	Lack of information (24, 51 %) No services (11, 23 %)
DPO	Lack of information (54, 61 %) No DPOs (21, 24 %)	Lack of information (14, 54 %) No DPOs (6, 23 %)
Education	Lack of information (47, 40 %) No services (28, 24 %)	Lack of information (29, 55 %) No services (14, 26 %)
Disaster management	Lack of information (29, 52 %) No disaster management in the area (16, 29 %)	Lack of information (16, 76 %) No disaster management in the area (3, 14 %)
Legal services	Lack of information (14, 56 %) No services (3, 12 %)	Lack of information (7, 50 %)

Note: Number of respondents considered is only those who reported unmet need and therefore the total number of respondents for each domain is different

## Discussion

This RAD survey has estimated the prevalence of disability at 6.8 (95 % CI: 5.9, 7.9) in Quezon City and 13.6 % (95 % CI: 11.4, 16.2) in Ligao City. These prevalence estimates are different from those derived from the 2010 Census of Population and Housing and the World Report on Disability because of differences in the methods used for measuring disability. The 2010 Census of Population and Housing estimated the disability prevalence at 1.6 % [10] using questions based on the short set of the Washington Group on Disability Statistics (WG) questionnaire [18]. The respondents were asked “Does ___ have any difficulty/problem in seeing, hearing, walking, remembering, self-care, and communication?” However, the respondents were asked to rate difficulty of functioning in the different domains on a dichotomous scale (yes/no) [10], which has been demonstrated to underestimate disability prevalence and may in fact correspond only to the prevalence of severe disability [19]. The World Report on Disability estimated the prevalence using an aggregate score measured from 15 questions in eight domains (vision, mobility, cognition, self-care, pain, interpersonal relationships, sleep, energy, and effect) from the World Health Survey (WHS) data (2002–2004) [1]. Each question asked for the level of difficulty on a 5-point Likert scale ranging from no difficulty to unable to do [1]. This estimate could be an overestimate because respondents were asked about difficulties with functioning in the 30 days prior to the interview and acute conditions might have been reported.

Prevalence estimates from the RAD survey are, however, closer to the estimates made by Mitra and Sambamoorthy who, using only questions related to difficulty in seeing, moving around, concentrating or remembering things, and self-care from 15 questions used in the WHS, estimated disability prevalence to be 8.3 % in the Philippines [20]. The similarity of their findings to results obtained using the RAD may be because both studies used questions about specific activity limitations. As demonstrated in the previous RAD survey in Bangladesh, prevalence estimates with RAD are comparable to the WG short set questions that are most widely used in Censuses. However, RAD also identifies respondents with psychological distress [5].

Consistent with other surveys [1, 3, 5, 20], this study also found the prevalence of disability is associated with increasing age and lack of schooling. As in other lower- and middle-income countries, rural-to-urban migration is common in the Philippines and people moving to urban areas are more likely to be young and educated [21]. In this survey, the sample from Quezon City was younger and had higher levels of education compared to the sample in Ligao City. The difference in the disability prevalence between the two sites was therefore not unexpected. It does, however, highlight the need to ensure health and rehabilitation services are available in rural areas, and have the capacity and systems in place to respond to the needs of older people who are more likely to have disability. There are several community-based rehabilitation programs in the Philippines and scaling up these programs to consider the needs of older people with disabilities would benefit communities with aging populations.

While psychological distress was not commonly identified in isolation, it was the most commonly reported difficulty in both locations. Psychosocial and emotional issues are not usually considered in disability data collection and people with psychosocial disability are often excluded from mainstream services and policies. Although the RAD survey focused only on psychological distress related to anxiety and depression, findings from this survey highlight that a large proportion of people with other types of disability experience psychological distress. There is a need for promoting counseling services and other support programs for people with psychosocial disability but also for those with other types of disability. Mental health is known to be associated with social participation, access to economic resources, and freedom from discrimination and violence, all conditions which people with disabilities are less likely to attain [22]. Therefore, in addition to responding to the psychological distress experienced by people with disabilities, it is important to address factors that contribute to their poor mental health when planning interventions.

Although women from both locations were more likely to have disability than men, the difference was statistically insignificant because of smaller sample size and limitation with recruiting participants in the survey. Although this survey had a good response rate, the majority of non-responders, particularly in Quezon City, were men either because they were away for work or because they did not want to participate in the survey. Some men declined to participate when they became aware that the survey related to disability, as issues related to household health, including disability, were perceived to be the responsibility of women. Household members and neighbors reported that many non-responders away from the home during the day were at work. We anticipate that, in the context of the Philippines, most people working outside the home would not have moderate or severe disability.

This survey did not find that household-level socioeconomic status based on the asset index was associated with disability. This finding is in contradiction with the current understanding of the relationship between poverty and disability [1, 3, 23, 24]. However, Loeb and Trani reported that disability and poverty as estimated using household asset index were not associated in Afghanistan and Zambia possibly because the majority of families surveyed in the two countries were poor [25]. Given that the majority of households in District 2 of Quezon City and Ligao City also live in impoverished conditions, we hypothesize this is why our survey also found no difference in disability prevalence for people from poorer and richer quintiles. Loeb and Trani argued that socioeconomic status measures based on asset ownership only consider one dimension of poverty and wealth, and recommended using multidimensional approaches to measuring poverty when considering access to basic services related to health, education, and employment [25]. Similar to their study, this survey also found that people with disabilities were deprived of access to these services compared to their age- and sex-matched controls.

This survey found that people with disabilities in the sample have poorer well-being and reduced access to services related to health, work, rehabilitation, education, government social welfare, and disaster management than people without disability. People with disabilities also experienced significant participation restrictions compared to people without disability. These findings are consistent with other surveys [1, 25, 26]. Barriers for participation reported by cases and controls were similar across most domains because the matched respondents belonged to communities that were poorly resourced. However, having a disability and negative family attitudes were reported as barriers for people with disabilities participating in work, community meetings, religious activities, and social activities suggesting stigma associated with disability in these communities.

### Strengths and limitations

One of the limitations of this survey was that the study areas were selected specifically because of the larger program, W-DARE. The study findings may not be generalized beyond the District 2 of Quezon City and Ligao City. In some of the particularly disadvantaged, urban areas non-responders could not be followed up by the field staff after working hours for security reasons. While this could potentially affect the generalizability of the findings, the impact may be insignificant because the response rate was good in both areas. As indicated earlier, the non-respondents are less likely to have a disability as the majority of them were away for work.

This study did not include children because the focus of W-DARE is adults and particularly women. Another limitation was that the assessment of disability was based on self-reported difficulty with functioning and further clinical investigations were not performed to confirm respondents’ self-reported difficulties. However, respondents who needed services were provided with appropriate referrals.

There were also a number of strengths to this study. Particularly, it was conducted in partnership with disabled people’s organizations and people with disabilities were part of the data collection team in both sites. This approach not only builds their capacity to conduct research, but demonstrates their capacity within their communities. This study provides reliable estimates of prevalence of disability, including measures of psychosocial distress and well-being, and allows for the understanding of the barriers to participation in community life experienced by people with disabilities in both areas. Understanding these factors is particularly important to inform the development of interventions in the latter phases of W-DARE. Comparing participation of people with disabilities with their age- and sex-matched controls is also important to inform future policies and programs in the study sites. As part of the participatory research process of W-DARE, findings have been disseminated to relevant government and non-government stakeholders in Quezon City and Ligao City emphasizing the importance of including people with disabilities in their policies and programs.

## Conclusion

The prevalence of disability among adults in District 2 of Quezon City and in Ligao City is higher than the estimated national prevalence of disability derived from the 2010 Philippines census. Disability is more prevalent among older respondents and those who had little or no education. Disability is also associated with reduced participation across a number of domains of community life. Our analysis highlights that psychological distress is common among people with disabilities. This is an important finding as psychosocial disability and psychological distress among people with other forms of disability are rarely measured and are therefore not addressed in national efforts to increase disability inclusion in low- and middle-income countries.

The input of Disabled People’s Organizations into our research approach and the inclusion of people with disabilities in the survey team has increased the capacity of researchers with disability in the Philippines, and increased engagement between people with disabilities and policymakers, service providers, and government representatives at all levels. This study has also generated considerable context-specific data, which the W-DARE project have used to inform the design of interventions to increase access to specific domains of community life for people with disabilities in Quezon City and Ligao City. Findings are relevant to government and other national agencies seeking to support universal access to a range of services, and more specifically, to respond to the needs of people with disabilities in the Philippines.

## Abbreviations

CI, Confidence interval; CRPD, Convention on the Rights of Persons with Disabilities; ICF, International Classification of Functioning, Disability and Health; RAD, Rapid Assessment of Disability; SD, Standard Deviation; SDGs, Global Goals for Sustainable Development; W-DARE Women with Disability taking Action on Reproductive and sexual health project; WHS, World Health Survey
